# Innovative Therapeutic Strategies for Effective Treatment of Brain Metastases

**DOI:** 10.3390/ijms20061280

**Published:** 2019-03-14

**Authors:** Malcolm Lim, Simon Puttick, Zachary H. Houston, Kristofer J. Thurecht, Priyakshi Kalita-de Croft, Stephen Mahler, Stephen E. Rose, Rosalind L. Jeffree, Roberta Mazzieri, Riccardo Dolcetti, Sunil R. Lakhani, Jodi M. Saunus

**Affiliations:** 1 Faculty of Medicine, the University of Queensland, Centre for Clinical Research, Herston, Queensland 4029, Australia; m.lim@uq.edu.au (M.L.); p.kalita@uq.edu.au (P.K.-d.C.); s.lakhani@uq.edu.au (S.R.L.); 2 Probing Biosystems Future Science Platform, CSIRO Health & Biosecurity, Herston, Queensland 4029, Australia; Simon.Puttick@csiro.au (S.P.); Stephen.Rose@csiro.au (S.E.R.); 3 Australian Institute for Bioengineering and Nanotechnology, the University of Queensland, St Lucia, Queensland 4072, Australia; z.houston@uq.edu.au (Z.H.H.); k.thurecht@uq.edu.au (K.J.T.); s.mahler@eng.uq.edu.au (S.M.); 4 Centre for Advanced Imaging, the University of Queensland, St Lucia, Queensland 4072, Australia; 5 ARC Centre of Excellence in Convergent BioNano Science and Technology, the University of Queensland, St Lucia, Queensland 4072, Australia; 6 ARC Training Centre for Biopharmaceutical Innovation, the University of Queensland, St Lucia, Queensland 4072, Australia; 7 Kenneth G. Jamieson Department of Neurosurgery, the Royal Brisbane and Women’s Hospital, Herston, Queensland 4029, Australia; Lindy.Jeffree@health.qld.gov.au; 8 Diamantina Institute, Translational Research Institute, the University of Queensland, Brisbane, Queensland 4102, Australia; r.mazzieri@uq.edu.au (R.M.); r.dolcetti@uq.edu.au (R.D.); 9 Pathology Queensland, the Royal Brisbane and Women’s Hospital, Herston, Queensland 4029, Australia

**Keywords:** brain metastases, nanomedicine, immunotherapy, drug delivery

## Abstract

Brain metastases are the most prevalent of intracranial malignancies. They are associated with a very poor prognosis and near 100% mortality. This has been the case for decades, largely because we lack effective therapeutics to augment surgery and radiotherapy. Notwithstanding improvements in the precision and efficacy of these life-prolonging treatments, with no reliable options for adjunct systemic therapy, brain recurrences are virtually inevitable. The factors limiting intracranial efficacy of existing agents are both physiological and molecular in nature. For example, heterogeneous permeability, abnormal perfusion and high interstitial pressure oppose the conventional convective delivery of circulating drugs, thus new delivery strategies are needed to achieve uniform drug uptake at therapeutic concentrations. Brain metastases are also highly adapted to their microenvironment, with complex cross-talk between the tumor, the stroma and the neural compartments driving speciation and drug resistance. New strategies must account for resistance mechanisms that are frequently engaged in this milieu, such as HER3 and other receptor tyrosine kinases that become induced and activated in the brain microenvironment. Here, we discuss molecular and physiological factors that contribute to the recalcitrance of these tumors, and review emerging therapeutic strategies, including agents targeting the PI3K axis, immunotherapies, nanomedicines and MRI-guided focused ultrasound for externally controlling drug delivery.

## 1. Introduction

The spread of cancer to the brain is a devastating complication associated with high morbidity and virtually 100% mortality. Brain metastases occur in 9–17% of cancer patients [1,2], most frequently in melanoma, lung and breast cancer [1,2]. They are broadly refractory to existing systemic therapies because of inefficient drug delivery, unique brain growth factors that promote resistance, and because they are usually detected at a late stage, when patients are already symptomatic. Solving these long-standing challenges around diagnosis and treatment requires innovation [3,4]. The risk of brain relapse for cancer patients today is higher than it was several decades ago [5,6,7]. In addition to population ageing, and improvements in the precision and accessibility of diagnostic imaging, this trend is also thought be a consequence of contemporary systemic drug therapies that are more effective against extracranial that intracranial cancer deposits. Consistent with this, adjuvant treatment of human epidermal growth factor receptor-2 positive (HER2+) breast cancer with the anti-HER2 monoclonal antibody (mAb) trastuzumab (Herceptin^®^) increases the likelihood that the brain will be the first site of symptomatic recurrence [8]. The aims of this review are to outline conventional management strategies (Section 2), pathophysiological and molecular factors that underpin the recalcitrance of brain metastases (Section 3) and review new approaches to systemic therapy where compelling preclinical and/or clinical evidence indicates they could play a role in clinical management in the future.

## 2. Current Management

Brain metastases are usually diagnosed by magnetic resonance imaging (MRI) in patients who present with neurological symptoms, such as headache, cognitive deficits or seizures. Depending on the number, sizes and location of lesions, age and general health, management may involve surgical resection, stereotactic or whole brain irradiation, with long-term steroid treatment to limit intracranial edema. Based on the widely-utilized Graded Prognostic Assessment (GPA) prognostic index, the median survival of patients with brain metastases is 7.16 months [9]. However, clinical outcomes vary considerably depending on primary cancer histology, comorbidities and the extent of metastatic disease elsewhere in the body. For example, HER2+ breast cancer patients with isolated brain metastases or synchronous but stable extracranial disease may live 5 years or more with multi-modal treatment, while those with triple-negative breast cancer (TNBC), non-small cell lung carcinoma (NSCLC) and melanoma typically succumb within 12 months, even with aggressive treatment.

### 2.1. Surgery and Radiotherapy

Surgery is most valuable for symptomatic lesions that are accessible, in patients with controlled extracranial disease and good performance status [10]. It has the advantage of relieving symptoms in 60–90% of patients [11,12,13], achieving local control in 60–100% [12,14], and enabling diagnostic testing for biomolecular markers, which may differ from the primary tumor; for example, expression of therapeutically targetable receptors for estrogen, progesterone and/or epidermal growth factor (ER/PR/HER2) is discordant in 20–50% of breast cancer cases [15,16]. Local recurrence after surgery is a persistent problem, occurring in 5–40% cases [10]. It can be reduced by resection of infiltrating cells beyond the gliotic capsule [12,14] and en bloc resection to avoid local “spillage” of malignant cells [10], but this not always safe or feasible, particularly in cystic or haemorrhagic metastases [17].

The addition of radiotherapy to surgery increases local control [10,18,19]. Historically, whole brain radiotherapy (WBRT) was used to try and also eliminate distant micrometastases, but randomized control trials showed no difference in survival [10] and there is evidence of delayed decline in cognitive function [20]. Consequently, many institutions currently offer post-operative focused stereotactic irradiation to the surgical resection cavity instead, although this has a higher risk of leptomeningeal or distant intracranial recurrence [18]. Radiotherapy, particularly stereotactic radiosurgery (SRS), may also be used as the primary treatment for local control, and is especially useful for deep, inaccessible lesions in patients with comorbidities. It is more effective for small tumors (<10 mm), and in certain tumor types, such as breast cancer, compared to melanoma and colorectal metastases [21,22,23]. When treating multiple tumors concurrently, the total tumor volume (<250 mm^3^) is more important than the number of metastases (up to 12) in achieving disease control [22,23]. Stereotactic irradiation can often be repeated if new lesions arise [22,24]. In spite of this, there are still too many patients whose brain metastases cannot be controlled by surgery or radiotherapy.

### 2.2. Systemic Therapy

In the setting of metastatic cancer with brain involvement, any survival benefit conferred by existing therapeutics is primarily through stabilization of extracranial disease, as opposed to measurable effects on brain metastases. However, exceptions to this generality are continually emerging, particularly molecular-targeted agents where there is diagnostic evidence of drug sensitivity. For example, erlotinib, gefitinib and the third-generation tyrosine kinase inhibitor (TKI) osimertinib are active in patients with EGFR-mutant NSCLC brain metastases [25] (particularly in combination with radiotherapy [26]), and there is also evidence that B-Raf (BRAF) and mitogen-activated protein kinase kinase (MEK) inhibition with dabrafenib plus trametinib treatment is active in BRAF-mutant melanoma brain metastases [27]. The challenge now is to use these agents in structured regimens to further improve patients’ longevity and maximize the arsenal of second- and subsequent-line therapies.

One reason for the slow development of effective treatments is an historic paucity of research investigating the molecular vulnerabilities and treatment barriers unique to brain metastases, using appropriate preclinical models and clinical samples [4]. Patients with symptomatic brain disease have been actively excluded from clinical trials because their prognosis is broadly incompatible with trial endpoints. Where they have been included, there was usually minimal or no impact on intracranial progression according to standard Response Evaluation Criteria in Solid Tumors (RECIST), which was widely interpreted to be a consequence of drugs not crossing an intact blood-brain-barrier (see below), and/or the disease being categorically chemoresistant. Both are assumptions we now know to be inaccurate, owing to a substantial increase in research activity over the last decade, and development of specific criteria to standardize the assessment of intracranial responses across trials [28]. This growing body of evidence suggests that to consistently achieve durable responses and good quality-of-life for our patients, we need compounds that target vulnerabilities specific to each cancer, with pharmacodynamic properties that overcome physiological drug delivery barriers at this site.

## 3. Why Are Brain Metastases Refractory to Conventional Treatment?

### 3.1. Protection of Micrometastases from Systemic Therapy

**Figure 1:**
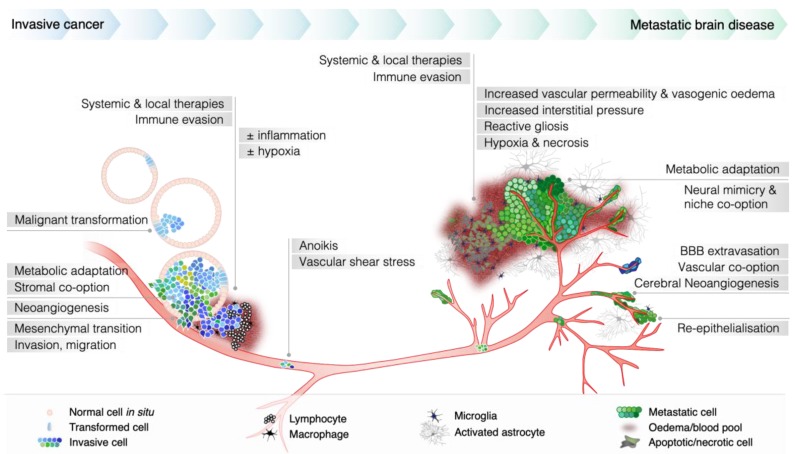
Schematic illustrating pathophysiological aspects of metastasis to the brain. Brain metastasis is an inefficient process marked by high rates of cellular attrition, and impacted by a myriad of selection pressures that drive clonal evolution. This includes requirements intrinsic to the metastatic cascade (capabilities for “metastatic fitness” are indicated in horizontal tracks) as well as extrinsic factors that drive the selection of particular tumor cell clones able to successfully establish brain metastases (vertical tracks; e.g., chemotherapy, radiotherapy or surgery) (adapted from [46]).

The pathophysiology of metastatic brain relapse is complex, impacted by a multitude of extrinsic selection pressures occurring over variable periods of time (Figure 1). The passage of molecules from the blood into the brain is tightly regulated by specialized blood vessels that perfuse the central nervous system (CNS). Tight junctions between vascular endothelia in this organ create a biological seal that effectively limits the diffusion of large molecules across paracellular clefts. Unlike peripheral blood vessels, this endothelium lacks fenestrations and only permits free diffusion of gases and small lipophilic molecules (<500 Da). Astrocyte foot processes further reinforce the endothelial layer via cellular communication and physical support [29,30]. Sandwiched between endothelia and foot-processes, pericytes and a basal lamina envelop the cerebral vasculature, where they regulate barrier function [31,32,33,34]. This specialized regulator complex is colloquially referred to as the blood-brain-barrier (BBB).

To colonise the brain, tumor cells must actively cross the BBB and evade local defences; or at least reach equilibrium with the ensuing inflammatory milieu. Evidence suggests they preferentially attach to endothelium that is inflamed and focally discontinuous, followed by enzymatic degradation of tight junctions and trans-endothelial migration [35,36]. Micrometastatic deposits remain dormant in the perivascular space for variable periods of time, protected from therapeutics and peripheral immune surveillance by the BBB for days, weeks or even years, until new adaptations (genomic, epigenetic and/or transcriptomic changes) tip the balance in favour of outgrowth. Astrocytes and microglia are involved, with tumor cell communication and growth factor co-option facilitating proliferation and immune avoidance [37,38,39,40]. Analysis of microenvironmental gene expression differences between brain metastases with low and high vascular permeability revealed that secretion of interleukin (IL)-6 and C-C motif chemokine ligand 2 (CCL2) by reactive (inflammatory) astrocytes contributes to BBB permeability [41]. Brain metastatic lung cancer cells can release IL-8, macrophage inhibitory factor, and plasminogen activator inhibitor-1, stimulating astrocytes to secrete tumor growth factors [42,43]. Tumor cells may also subvert astrocytic attack by producing serpins [44,45].

### 3.2. Microenvironmental Adaptation and Clonal Selection

**Figure 2:**
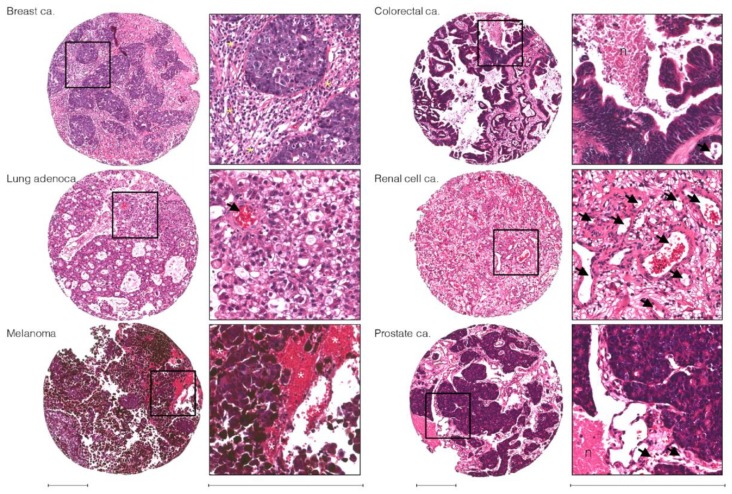
Typical histological appearance of brain metastases from different primary tumor types. Hematoxylin and eosin (H&E)-stained tissue sections show that brain metastases usually exhibit morphology typical of the primary tumor of origin. Features often seen in brain metastasis surgical specimens are highlighted: intact and dilated blood vessels (arrows; high microvascular density in the renal cell carcinoma-brain metastasis shown), hemorrhagic regions typical of melanoma brain metastases (white asterisks), necrotic tissue (“n”) and reactive gliosis around tumor cell nests with pushing margins (yellow asterisks). Scale bars = 250 μm. ca., carcinoma; adenoca. adenocarcinoma.

Cells that successfully invade the perivascular space and survive seem to migrate along paths of least resistance. Some proliferate in the perivascular pathway, forming sheaths that co-opt the vessels; while others take interstitial tracks and form well-demarcated parenchymal lesions that presumably depend more on neo-angiogenesis for a constant supply of metabolic substrates. The degree to which these mechanisms are used varies according to tumor histology. For example, melanoma brain metastases are highly vascular and tend to grow as haemorrhagic, perivascular proliferations, while carcinomas are more likely to develop in the parenchyma with pushing margins [47,48,49] (Figure 2).

Tumor cells use remarkable mechanisms to co-opt the neural niche. There is evidence that they can repurpose neurotransmitters as metabolic substrates, recruit and promote the differentiation of neural progenitors into tumor-supporting reactive astrocytes, and mimic neural traits to effectively “plug-and-play” with the new soil. Brain growth factors play critical roles in driving speciation of cancer cells and development of drug resistance, particularly mechanisms that promote phosphoinositide 3-kinase (PI3K) activity [3,50,51,52,53,54]. Molecular profiling of surgical samples has identified frequent activating alterations in *PIK3CA*, amplification and overexpression of upstream growth factor receptors (e.g., HER2, HER3, c-MET [55,56,57,58]) and repression of its suppressor, phosphatase and tensin homolog (PTEN), through genomic loss, inactivating mutation or promoter methylation [57,59,60,61]. Post-transcriptional silencing has also been identified as a mechanism suppressing PTEN in brain metastases from breast cancer, resulting from the delivery of *PTEN*-specific miRNAs to tumor cells by re-educated astrocytes in the tumor microenvironment [62]. These studies and others collectively indicated that inhibiting the PI3K/Akt/mTOR pathway is a promising strategy for treatment. 

Comparing patient-matched pairs of primary and metastatic tumor tissue can reveal differences that arise from selective pressure imposed by treatment and/or the microenvironment [57,60,63,64]. Brastianos and colleagues sequenced the exomes of 86 trios for deep analysis of clonal selection: primary breast tumors, metastases (mostly brain, including a handful of cases with sequential brain tumors) and blood as a source of germline DNA [60]. The majority of cases exhibited branching evolution, with a “trunk” of shared changes and a series of “private” mutations reflecting continual independent evolution. Alterations predicting sensitivity to PI3K/AKT/mTOR, cyclin-dependent kinases (CDKs) and HER2/EGFR inhibitors were identified; and importantly, in 53% of patients, clinically informative mutations were not detected in the primary tumor tissue sample. While extracranial metastases may be more accessible for biopsy than brain metastases, they were found to be highly divergent in this study. These and related studies indicate that ideally, management decisions should be based on direct diagnostic profiling wherever possible, rather than information inferred from analysis of the primary tumor or other metastases. Although it is recommended that metastatic breast cancer treatment be based on the biomarker profiles of available metastases [65], brain tumor biopsy is often challenging or not possible. There is a clear unmet need for less invasive modalities to measure biomarker expression in metastatic brain lesions.

Treatment can also be a key driver of extrinsically driven clonal evolution in brain metastases. For example, radiotherapy is a critical component of clinical management that on balance, is effective in slowing relapse and improving quality-of-life via debulking. However, there is also evidence that it promotes progression in hypoxic tumor niches [66], where an accumulation of hypoxia-inducible factor-1 alpha (HIF1-α) has likely already primed cells for survival and tissue regeneration. A local deficiency of molecular oxygen can limit the extent of DNA damage caused by ionizing radiation, expediting clonal evolution rather than the intended effect of catastrophic genome instability and apoptosis. Paradoxically, hypoxia also characterizes regions of post-treatment radiation necrosis [67], and thus overall, is a potent barrier to effective radiotherapy in brain and other solid tumors [68,69,70]. Preclinical studies have implicated c-Met signaling in hypoxia- and radiotherapy-associated protection from apoptosis [71,72]. Supporting this, two case studies recently reported slower disease progression in brain metastasis patients treated with c-Met inhibitors, crizotinib or cabozantinib [73,74].

### 3.3. Abnormal Perfusion Dynamics and High Tumor Interstitial Pressure

In established, symptomatic lesions, an abnormal capillary network that evolves in parallel with the tumor takes over from the BBB as the chief opponent of drug delivery. Brain metastases are characterized by perpetual, spatially overlapping cycles of proliferation, localized hypoxia and angiogenesis. The tips of sprouting vessels are intrinsically “leaky” because the immature BBB is devoid of tight junctions [3,75], and continual vascular remodelling produces a tortuous, dysfunctional vascular bed with heterogeneous, but overall elevated permeability. The crucial, detrimental consequence is that this abolishes the pressure gradient between the vascular compartment and surrounding brain tissue, resulting in edema and locally elevated interstitial fluid pressure that physically oppose penetration of circulating compounds into the tumor. Moreover, blood flow becomes largely static, exacerbating hypoxia and poor drug accumulation in an environment that is, paradoxically, highly vascularized [76,77,78,79]. Particular to brain metastases is that this occurs in the closed cranial cavity, which has little biophysical capacity to diffuse additional fluid pressure. Therefore, established, symptomatic brain metastases present unique barriers to simple convective uptake and retention of circulating drugs [80].

### 3.4. Late Clinical Detection

A critical outstanding question is whether earlier diagnosis and treatment of brain metastases would materially improve clinical outcomes. Currently, brain imaging is not routinely performed after treatment for primary cancer unless there is a specific indication or new symptoms warranting investigation. The decision to not perform ongoing surveillance for metastatic disease is largely economic, but also clinical, as conventional MRI can detect abnormalities of unknown significance, prompting repeat scanning or biopsy for a definitive diagnosis. This presents the potential to cause physical harm, and also psychological harm due to an uncertain diagnosis, or a diagnosis with no actionable treatment plan. Even if surveillance was routinely conducted in high-risk patients, by the time brain metastases are detected or symptomatic they are already resistant to multiple lines of therapy and are equipped for efficient adaptation to new selection pressures. So long as they are identified at this late stage, treatment benefits will continue to be incremental [3].

If detection and treatment of small, asymptomatic lesions is to be considered, we also need diagnostic tools with precision and specificity that exceed the standard achievable with diagnostic MRI. With significant research efforts being directed towards the application of machine learning methods in image analysis [81,82], it is becoming possible to correlate more subtle image parameters with the development of brain metastases [83,84]. However, simultaneous acquisition of positron-emission tomography (PET)-based tumor biomarker information will likely be required to achieve the specificity and sensitivity required for clinical implementation as a surveillance modality.

## 4. Innovative Strategies to Overcome Treatment Barriers Unique to Brain Metastases

**Figure 3:**
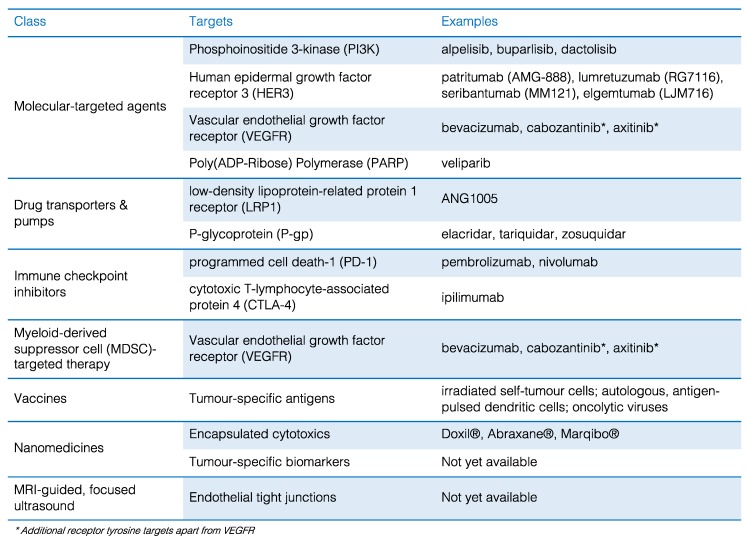
Summary of the approaches discussed in Section 4, including therapeutic classes, their molecular targets and examples of experimental or clinically approved agents.

Considering the combination of pathophysiologic and clinical factors limiting the delivery and efficacy of systemic therapy in brain metastases, it is clear that development of more effective strategies will require significant investment in innovative research that crosses disciplinary boundaries. This section will review emerging approaches that may be able to overcome some of the current limitations, and/or have strong clinical momentum in this setting (Figure 3).

### 4.1. New Molecular-Targeted Agents

The PI3K/Akt/mTOR signaling pathway is a potent mediator of cell survival and drug resistance, and a key driver of brain metastasis [62,85,86,87], with more than 50% of breast and lung cancer brain metastases harboring *PIK3CA* mutations or genomic amplification [57,60]. PI3K inhibitors including alpelisib, buparlisib and dactolisib are currently being evaluated in the setting of advanced cancer where there is diagnostic evidence of mutation. In the SOLAR-1 registrational trial, alpelisib reduced the risk of progression or death by 35% in patients with ER+, HER2-negative, PIK3CA-mutant advanced breast cancer [88], but assessment of CNS progression is currently not planned. On the other hand, buparlisib is brain-penetrant, inhibits intracranial outgrowth in preclinical models, was well-tolerated in phase-I studies and reduced metastatic brain tumor volume by 28% in one patient [89,90,91]. It has also been evaluated in ER+, HER2-negative advanced breast cancer, and in both trials increased progression-free survival, but produced significant toxicity [92,93]. This side-effect profile could be problematic in patients with brain involvement, and thus a carefully designed phase-II trial is required to assess overall clinical benefit and identify additional predictive markers to help further refine the treatment population.

Kodack and colleagues recently demonstrated that HER3 induction was an effective escape mechanism for experimental brain metastases treated with buparlisib, but that sensitivity could be restored by simultaneously treating mice with a HER3-targeted mAb [87]. Since HER3 induction and activation are common in brain metastases from a multitude of cancers [55,57,58], combination strategies with HER3 inhibition may be more effective than PI3K monotherapy. The central role of HER3 in chemotherapeutic resistance has led to development of HER3-targeted monoclonal antibodies (mAbs), which are being clinically evaluated in various solid cancers [94,95]; for example, patritumab (AMG-888; Daiichi-Sankyo), lumretuzumab (RG7116; Roche), seribantumab (MM121; Merrimack), elgemtumab (LJM716; Novartis). HER3 is induced in brain metastases compared to matching breast and lung primary tumors, is frequently phosphorylated in brain metastases with various histologies, and in primary breast cancers, its overexpression increases the likelihood that the brain will be the first site of relapse [55,58,96,97]. Activation of PI3K/MAPK signaling through HER3 could also explain resistance to other targeted agents in intracranial versus extracranial metastases [98,99]. Therefore, there is accumulating evidence in favor of trialing anti-HER3 therapy in a variety of contexts for cancer patients with brain involvement.

Targeting vascular endothelial growth factor (VEGF) activity has emerged as another promising strategy that acts largely by suppressing neo-vascularization and normalizing perfusion in hyperpermeable tumors. The VEGF-receptor inhibitor (VEGFRi), bevacizumab (Avastin^®^), has limited activity as a single agent, but improves delivery and potentiates the effects of others [77,100]. At least 22 phase-II clinical trials are ongoing to test the clinical benefit of combining bevacizumab with other agents for patients with brain metastases [101]. The VEGFR2i, cabozantinib, is also being evaluated in combination with trastuzumab in HER2+ breast cancer patients with brain metastases (NCT02260531) on the basis of its broader range of tyrosine kinase receptor targets, including c-MET, RET and AXL. In addition to directly improving oxygenation and drug delivery in solid tumors, VEGFR inhibition may also enhance anti-tumor immunity, as VEGF has a multitude of immunosuppressive effects, including direct action on immune cells ([102], see Section 4.3).

Poly(ADP-Ribose) Polymerase inhibitors (PARPi) represent a new class of agents that exploit dsDNA break repair defects and addiction to alternative, PARP-mediated DNA repair in cancer cells. This genetic theory of “synthetic lethality” has been validated in breast cancer patients with germline loss-of-function mutations in BRCA1 or BRCA2. Research identifying ways to maximize clinical effectiveness is still underway, with potential approaches being to give PARPi as chemo- or radio-sensitizing agents. Genome instability and BRCA1 dysfunction characterize some breast cancer brain metastases, and may predict benefit from PARPi [103,104]. The PARPi veliparib was investigated in combination with WBRT in brain metastases from solid tumors. Results from the phase-1 dose-escalation study were encouraging, but the second-phase randomized trial comparing WBRT with and without veliparib in NSCLC brain metastases revealed no significant differences in disease progression between treatment groups [105,106]. It is currently being investigated in combination with cisplatin in TNBC or BRCA-associated breast cancer with brain metastases (NCT02595905). A phase 2 trial of iniparib with irinotecan to treat patient brain metastatic TNBC reported modest survival benefit [107]. It is not clear whether this outcome is attributable to PARP inhibition directly, and/or an increase in reactive oxygen species [108].

### 4.2. Drug-BBB Transporter Conjugates

The selective passage of nutrients and metabolites essential for neuronal function from the bloodstream to the brain parenchyma is regulated by transporter proteins that are differentially expressed on the luminal and abluminal membranes of brain endothelia. In an attempt to increase drug delivery to the CNS, innovative therapeutic strategies are being developed that exploit three major BBB transcytosis pathways:

1. Adsorptive transcytosis occurs when cationic molecules bind to negatively-charged clathrin-coated or caveolar vesicles on brain endothelial cells. Drug delivery strategies harnessing this pathway involve direct chemical modifications; such as cationization of therapeutic molecules, which promotes adhesion to the anionic cell membrane [109]. However, since anionic sites are found in all living cells, the risk of off-target drug toxicity is significant [110].
2. Carrier-mediated transporters specifically translocate small molecule nutrients such as glucose, amino acids, amines, nucleosides and small peptides. Drug targeting using carrier-mediated transport involves conjugation to its endogenous substrate or mimic ligand. Glucose transporters are highly expressed by the BBB, are well-characterized and have evolved to meet the metabolic demands of the brain, making them good drug delivery vehicle candidates. One study reported that the density of ligands decorating the conjugate correlated with blood-brain transport efficiency [111]. Liposomes coated with a high density of the glucose derivative glycosyl achieved close to 3-fold higher brain uptake than unconjugated liposomes or liposomes with low glycosyl density [111]. Supporting this, glucose-coated paclitaxel nanoparticles were effective in a mouse model of glioma [112].
3. Receptor-mediated drug transport strategies rely on molecular ligand mimicry to induce endocytosis and transport to the abluminal membrane opposing the brain parenchyma. Referred as the “trojan horse”, this approach has garnered more attention in the field than the former, because of the potential for ferrying larger cargoes conjugated to molecules like transferrin and insulin. These conjugates have been developed and successfully applied in neurological conditions; for example, the large neutral amino acid transporter (LAT1) has been exploited to transport gabapentin in epilepsy [113], L-DOPA in Parkinson’s disease [114] and baclofen for patients with cerebral palsy [115]. Another example is a bispecific antibody against the transferrin receptor (TfR) and β-secretase, an Alzheimer’s disease drug target developed by Genentech in 2011 [116]. Interestingly, high-affinity monoclonal antibody (mAb) resulted either in the conjugate being sorted to lysosomal degradation or remaining trapped to the receptor after abluminal trafficking, resulting in TfR deficiency in the brain. On the other hand, a lower affinity mAb transcytosed and successfully dissociated from TfR, resulting in more drug uptake in the brain parenchyma and no associated neurotoxicity [116,117].

BBB transporter-taxol conjugates are also beginning to show potential for treating brain tumors. ANG1005 comprises paclitaxel linked to Angiopep-2, a peptide ligand for low-density lipoprotein-related protein 1 receptor, which is highly expressed on BBB endothelia. Preclinical studies showed that ANG1005 significantly prolonged the survival of mice after intracerebral injection with NSCLC cells [118]. Further, a mouse brain perfusion study indicated that ANG1005 also bypassed p-glycoprotein-mediated efflux, leading to greater uptake compared to unconjugated paclitaxel [118]. Clinically, it produced survival benefit in patients with both primary and secondary brain cancers [119,120,121,122], was granted orphan drug status by the U.S. Food and Drug Administration (FDA) for treating the aggressive primary brain tumor glioblastoma (GBM) in 2014.

Preclinical and clinical studies continue to be invaluable for developing a better understanding of the caveats of receptor-mediated drug transport, highlighting several key pharmacodynamic considerations, including competition with endogenous substrates, ligand-binding affinity, effect on normal receptor trafficking and interaction with xenobiotic efflux pumps like P-glycoprotein (P-gp; encoded by ATP-binding cassette sub-family B member 1 (ABCB1)) and Breast Cancer Resistance Protein (BCRP; *ABCG2*). Preclinical studies with the P-gp inhibitors elacridar, tariquidar and zosuquidar hinted at the possibility that inhibiting efflux could improve the efficacy of other treatments in the brain [123,124,125]. However, the pharmacokinetic profile of P-gp inhibitors presents significant challenges to clinical translation, related to non-specific interaction with cytochrome P450 enzymes, competition for target binding with other therapeutics and the ubiquitous expression of efflux pumps throughout the body. Several clinical studies reported increased toxicity in patients treated with P-gp inhibitors [126,127,128,129], and so far, improvement in clinical outcomes of patients with solid tumors have been mediocre [128,130,131].

### 4.3. Immunotherapy

The brain is an immune-specialized site with unique defenses. It is protected by an independent innate immune system comprising glia, ependymal cells and neurons, all capable of antigen presentation [132,133,134]. Brain-resident macrophages (microglia) monitor the microenvironment and react to non-self-antigens by adopting an inflammatory phenotype, involving increased motility, phagocytosis and proliferation. Reactive astrocytes also participate by expressing inflammatory mediators and exhibiting phagocytic activity [135,136,137]. Though devoid of typical lymphoid tissue, the brain has specialized lymphatic vessels that drain antigens and antigen-presenting cells (APCs) from the dural sinuses to cervical lymph node chains [138], where they are capable of inducing responses that involve infiltration of the brain parenchyma by peripheral effector cells via the BBB, choroid plexus epithelium and glia limitans [139,140,141,142].

#### 4.3.1. Immune Checkpoint Inhibitors

T-cells become reactive when the T-cell receptor binds an antigen presented by APCs via the major histocompatibility complex (MHC), with appropriate “danger”-induced co-stimulatory molecules. If a T-cell simultaneously binds a co-inhibitory ligand (“negative checkpoint”), this impairs the T-cell response. Inhibitory checkpoint activation is a physiological negative feedback mechanism to avoid excessive reactivity and maintain self-tolerance. It is strongly promoted by chronic exposure of activated effector cells to tumor antigens and the immunosuppressive microenvironments of advanced cancers, and is considered to be a key driver of immune evasion during progression. Targeting checkpoints that constrain anti-tumor immune responses has emerged as a revolutionary approach allowing long-term survival of a consistent proportion of patients with disseminated cancer. Great efforts are being made to develop co-stimulatory receptor agonists (e.g., OX40, 4-1BB) and inhibitory receptor antagonists (e.g., CTLA-4, PD-1/PD-L1, VISTA, LAG-3, TIM-3) to restore and/or reinvigorate anti-tumor T-cell responses. In the clinical setting, immune checkpoint inhibitors (ICIs) can trigger clinically relevant anti-tumor responses not only in classically immunogenic tumor types like melanoma, but also other solid tumors.

New findings have revealed that ICIs can also produce durable effects against brain metastases—the first truly optimistic results for this patient population in decades [143,144,145,146,147]. For example, intracranial response rates of 20–30% were observed in melanoma and NSCLC patients treated with pembrolizumab (anti-programmed cell death-1 (PD-1)), and nivolumab plus ipilimumab (anti-PD1 and -cytotoxic T-lymphocyte-associated protein 4 (CTLA-4)) produced objective CNS responses in 55% of melanoma patients [148,149]. Interestingly, preclinical studies suggest that ICIs are most effective against brain metastases when there is concurrent extracranial disease. Intracranial responses were attributed to a substantial increase in CD8+ T-cell trafficking into the brain after expansion of homing-competent T-cells in the periphery, potentiated by induction of T-cell entry receptors on brain microvasculature [150]. The field eagerly awaits results from trials investigating the benefit of combining ICIs with chemo- and radiotherapy, which enhance the production of neoantigens that can subsequently stimulate anti-tumor immunity. The initial results available seem promising, as shown by a recent report in metastatic melanoma, where concurrent SRS and ICI therapy improved intracranial control and survival compared with historical controls patients [151].

#### 4.3.2. Myeloid-Derived Suppressor Cell (MDSC)-Targeted Therapy

In cancer and other chronic inflammatory conditions, the differentiation of immature myeloid cells becomes biased toward expansion of myeloid-derived suppressor cells (MDSCs) at the expense of the monocyte and neutrophil populations [152]. MDSCs promote cancer progression in multiple ways, including active suppression of T lymphocytes and Natural Killer cells, and as such they are a key target of interest in oncology. A reduction in absolute numbers and function of MDSCs correlate with the efficacy of VEGF-targeted therapy, and recent findings have implicated this mechanism in intracranial activity of VEGFRi. For example, bevacizumab significantly delayed intracranial progression amongst stage-IV EGFR-mutant lung cancer patients, associated with a higher ratio of effector T-cells to peripheral MDSCs, and pre-treatment MDSC levels correlated with progression-free survival in this trial [153]. Likewise, axitinib prolonged survival of experimental mice bearing melanoma-brain xenografts, and this was associated with MDSC differentiation toward an antigen-presenting phenotype with reduced suppressor activity [154]. More clinical studies are warranted to investigate the optimal contexts for MDSC-targeted approaches in brain metastatic cancer. Notably, there are currently no clinically approved agents that directly target this cell population.

#### 4.3.3. Cancer Vaccines

A vaccine is a therapy targeted at acquiring long-term immunity. Expressed, somatically mutated genes sometimes encode neoantigens that are potentially immunogenic and therapeutically targetable, but clinical implementation of an optimized process for identifying, validating and developing such precision vaccines remains complex. Contrary to early expectations, overexpressed self-antigens, particularly those with direct roles in tumorigenesis, can be highly immunogenic, and in fact comprise the largest class of candidate targets for vaccine development [155]. In contrast to neoantigen vaccination strategies, this class is applicable to larger patient groups and is amenable to development of an “off-the-shelf” suite of cancer vaccines that could be combined in personalized, polyvalent cancer vaccines. PERCELLVAC3 (NCT02808416) is an ongoing phase I/II trial evaluating the safety and efficacy of personalized cellular vaccines in patients with brain metastases. Patients are immunized with autologous, irradiated tumor cells, or APCs that have been pulsed with tumor antigen-encoding RNA identified by profiling tumor material after surgery.

Oncolytic viruses that selectively infect tumor cells are emerging as a novel class of cancer immuno-therapeutics that may be considered a particular form of vaccination against cancer. In fact, oncolytic viruses induce immune responses not only against viral antigens but also against tumor antigens and can thereby also target non-infected metastases. To overcome the hurdles of systemic delivery, it has been proposed to arm mesenchymal stem cells with oncolytic viruses. In a preclinical model, injection of these cells in combination with PD-L1 blockade increased interferon gamma (IFNγ)-producing CD8+ tumor-infiltrating T lymphocytes and significantly prolonged survival [156].

#### 4.3.4. Clinical Considerations for Treating Brain Metastases with Immune-Modulating Therapies

Used to limit peritumoral edema and intracranial pressure, dexamethasone is a core component of management for brain metastasis patients. But corticosteroids like dexamethasone are also potent immunosuppressants that counteract the effects of immunotherapy. In contrast to asymptomatic melanoma patients with brain metastases, ipilimumab conferred no survival benefit to patients being managed with corticosteroids [145]. Furthermore, other melanoma and lung cancer trials also reported ICIs were more effective when prednisone or dexamethasone were not required for symptom control [157,158]. Ideally, ICIs should be given before corticosteroids, which would require diagnosis before the onset of neurological symptoms, and so the promise of immunotherapy poses a new rationale for earlier detection of smaller lesions through surveillance of high-risk patients. In general, the main limitation of ICIs is the possibility of T-cell activation causing an adverse immune reaction, but there are additional risks associated with therapeutically stimulating immune responses within the cranial cavity, where sharp increases in pressure from cytokine storming and edema could be life-threatening. However, the incidence of grade-3/4 adverse neurological events in early GBM and brain metastatic melanoma trials was not particularly frequent (~7% of cases); considered to be acceptable in these groups that already have a very poor prognosis.

### 4.4. Nanomedicines

Studies from the 1970s–90s reported intracranial responses in up to 59% of metastatic breast cancer patients treated with cytotoxic chemotherapy, including cyclophosphamide, doxorubicin, vincristine, methotrexate and 5-fluorouracil [159,160]. Mechanistically, this makes sense as brain metastases exhibit rapid proliferation and genomic instability—hallmarks of chemosensitivity [161]. In current practice, chemotherapy is usually prescribed secondary to local treatment, and/or with the aim of stabilizing extracranial disease, with poor CNS penetration and drug resistance often cited as the rationale. However, in early trials, intracranial effects paralleled systemic responses [159,160], consistent with the notion that the sensitivity of the underlying tumor histology to a particular therapy is more important than its apparent CNS penetrance [162]. It has also been challenging to exploit such intrinsic vulnerabilities in this patient population, where the comorbidity burden is high and the therapeutic indices of most cytotoxics are already narrow.

**Figure 4:**
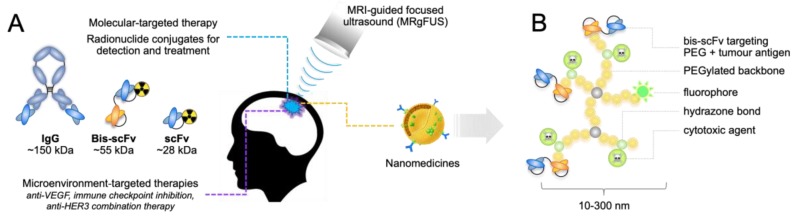
Emerging strategies for detection and treatment of brain metastases. (**A**) Bioengineering and molecular oncology research are beginning to identify approaches that specifically target vulnerabilities of brain metastases, including microenvironmental adaptations. Through flexibility of design, nanomedicine approaches offer exciting possibilities for overcoming drug delivery and therapeutic index challenges in the metastatic brain cancer patient population. Externally-augmented drug release and/or activation may be a useful therapeutic tool in some circumstances. (**B**) Typical structure of a polymer-based nanocarrier with various functional groups. bis-scFv, bispecific single-chain variable fragment; IgG, immunoglobulin; kDa, kilodalton; nm, nanometer.

The emergent nanomedicine field offers possibilities to circumvent toxicity through drug design. Nanomedicine is the application of nanotechnology in medicine to diagnose, prevent and treat disease. Integrating bioengineering with immuno/oncology and nuclear medicine is an exciting prospect for cancer management; particularly brain tumors, which present unique drug delivery challenges and where there is great opportunity to positively impact patient outcomes [4]. Key design features include (Figure 4):

1. Shell material to enhance solubility and delay clearance by minimizing immune recognition. For example, polyethylene glycol (PEG) minimizes recognition by mononuclear phagocytes. PEGylation also reportedly increased distribution of cytotoxic drugs in the brain and provided resistance to enzymatic decay [163,164,165].
2. Molecular targeting functionality, generally via conjugated mAbs or mAb fragments. This is achieved using a number of approaches, including engineered proteins with dual specificity to tether the shell material to tumor antigens [166].

Using this technology, therapeutics with proven efficacy in particular settings can be redesigned to mitigate physicochemical, biological and physiological barriers, targeting payloads with anatomic and molecular precision [167,168,169,170]. This can improve pharmaco-kinetic and -dynamic profiles, increase drug accumulation and specificity, and reduce systemic toxicity. At least 50 nanocarriers have been clinically approved (e.g., Doxil^®^, Abraxane^®^, Marqibo^®^), with another 80 or so being tested in ongoing trials [171]. Most are relatively simple conjugates, but translational research efforts are now focusing on parallel diagnostic and therapeutic applications for bespoke carriers. With further development, patients at high risk of brain relapse could be administered with tumor-targeted carriers loaded with a PET isotope, then if disease is detected in the brain using diagnostic PET-MRI, administered with equivalent carriers carrying a therapeutic payload. This approach could also allow personalized, early treatment without the need for a tissue biopsy. The tunability, synthetic flexibility, and longer circulatory half-lives of nanomedicines offer significant potential in precision medicine, including the treatment or prevention of recurrent brain tumors.

### 4.5. MRI-Guided, Focused Ultrasound

The BBB presents a significant challenge for delivering systemic agents to brain micrometastases and regions of established tumors where it is still intact. Remodelling the tumor vasculature using MRI-guided, focused ultrasound (MRgFUS) is showing promise for brain tumor management [172,173]. Gas-filled, lipid microspheres (microbubbles, MB) have been used in echocardiography for many years but more recently it has been shown that in combination with MRgFUS, they disrupt tumor vasculature and increase drug delivery [174]. Due to the mismatch in acoustic impedance between their gaseous cores and surrounding tissue, MBs resonate when excited by ultrasound. Under low acoustic pressure, MBs undergo repeated expansion and contraction, (cavitation) which generates flow around their surfaces (microstreaming), causes shear stress to cell membranes in the vicinity and disruption of endothelial tight junctions [175,176]. This transiently increases vascular permeability and the uptake of circulating compounds, including engineered nanomaterials [174].

Arvantis and colleagues found that MRgFUS increased the uptake of trastuzumab, the mAb-cytotoxic conjugate, ado-trastuzumab emtansine (T-DM1) and doxorubicin in experimental brain metastases by 2–7-fold [177]. Pharmacokinetic modelling indicated a virtually linear increase in transvascular flux through BBB pores up to 50 nm in diameter, theoretically permissive to small nanoparticles. Preclinical GBM modelling has shown 2-fold higher uptake of paclitaxel-loaded liposomes of 120 nm diameter after administering MBs with FUS [178], though generally, smaller particles are likely to be most effective due not only to BBB pore size limitations, but also susceptibility to interstitial hypertension during the post-extravasation phase, where movement through the extracellular matrix is largely via passive diffusion [179,180,181]. Although still an investigational procedure, a recent study in patients with malignant glioma suggests that MRgFUS is safe, feasible, and can increase the concentration of systemically delivered chemotherapy by temporarily disrupting the BBB [182].

## 5. Concluding Remarks

This review illustrates the importance of pharmacodynamic and pathophysiological context to the systemic treatment of metastatic brain disease. Historically, interpretation of clinical trials failed to adequately consider the pathophysiological context of each treatment, and there was a disproportionate focus on the BBB as an immutable obstacle to effective systemic therapy. However, it has become clear that the barriers to successful treatment are different in early and later stages of brain metastasis development [46], and it is crucial that future designs establish the disease entity being targeted to ensure success. From a biological point-of-view, the goals of immuno-/oncologic therapy could be broadly divided as follows:

1. Specifically target subclinical deposits with an intact BBB:
  - to prevent outgrowth in patients at high risk of brain relapse who are undergoing first-line treatment. This has the greatest potential to impact clinical outcomes, but requires accurate risk prediction protocols;
  - as an adjunct to local treatment to limit post-treatment recurrence, or new recurrences that arise from “self-seeding”.
2. Specifically target established, symptomatic tumors that are not suitable for surgery or radiotherapy using innovative drug conjugates and combination approaches to overcome physiologic barriers to the simple convective delivery.

## References

[1] Nayak L., Lee E.Q., Wen P.Y. (2012). Epidemiology of brain metastases. Curr. Oncol. Rep..

[2] Australia Institute of Health and Welfare (2017). Cancer in Australia 2017.

[3] Kodack D.P., Askoxylakis V., Ferraro G.B., Fukumura D., Jain R.K. (2015). Emerging Strategies for Treating Brain Metastases from Breast Cancer. Cancer Cell.

[4] Maher E.A., Mietz J., Arteaga C.L., DePinho R.A., Mohla S. (2009). Brain metastasis: Opportunities in basic and translational research. Cancer Res..

[5] Smedby K.E., Brandt L., Backlund M.L., Blomqvist P. (2009). Brain metastases admissions in Sweden between 1987 and 2006. Br. J. Cancer.

[6] Frisk G., Svensson T., Backlund L.M., Lidbrink E., Blomqvist P., Smedby K.E. (2012). Incidence and time trends of brain metastases admissions among breast cancer patients in Sweden. Br. J. Cancer.

[7] Pelletier E.M., Shim B., Goodman S., Amonkar M.M. (2008). Epidemiology and economic burden of brain metastases among patients with primary breast cancer: Results from a US claims data analysis. Breast Cancer Res. Treat..

[8] Olson E.M., Abdel-Rasoul M., Maly J., Wu C.S., Lin N.U., Shapiro C.L. (2013). Incidence and risk of central nervous system metastases as site of first recurrence in patients with HER2-positive breast cancer treated with adjuvant trastuzumab. Ann. Oncol..

[9] Sperduto P.W., Kased N., Roberge D., Xu Z., Shanley R., Luo X., Sneed P.K., Chao S.T., Weil R.J., Suh J. (2012). Summary report on the graded prognostic assessment: An accurate and facile diagnosis-specific tool to estimate survival for patients with brain metastases. J. Clin. Oncol. Off. J. Am. Soc. Clin. Oncol..

[10] Siu T.L., Jeffree R.L., Fuller J.W. (2011). Current strategies in the surgical management of cerebral metastases: An evidence-based review. J. Clin. Neurosci..

[11] Bartsch R., Berghoff A.S., Preusser M. (2013). Optimal management of brain metastases from breast cancer. Issues and considerations. CNS Drugs.

[12] Pessina F., Navarria P., Cozzi L., Ascolese A.M., Maggi G., Rossi M., Riva M., Scorsetti M., Bello L. (2016). Role of Surgical Resection in Patients with Single Large Brain Metastases: Feasibility, Morbidity, and Local Control Evaluation. World Neurosurg..

[13] Salvati M., Tropeano M.P., Maiola V., Lavalle L., Brogna C., Colonnese C., Frati A., D’Elia A. (2018). Multiple brain metastases: A surgical series and neurosurgical perspective. Neurol. Sci..

[14] Yoo H., Kim Y.Z., Nam B.H., Shin S.H., Yang H.S., Lee J.S., Zo J.I., Lee S.H. (2009). Reduced local recurrence of a single brain metastasis through microscopic total resection. J. Neurosurg..

[15] Jung J., Lee S.H., Park M., Youn J.H., Shin S.H., Gwak H.S., Yoo H. (2018). Discordances in ER, PR, and HER2 between primary breast cancer and brain metastasis. J. Neurooncol..

[16] Kaidar-Person O., Meattini I., Jain P., Bult P., Simone N., Kindts I., Steffens R., Weltens C., Navarria P., Belkacemi Y. (2018). Discrepancies between biomarkers of primary breast cancer and subsequent brain metastases: An international multicenter study. Breast Cancer Res. Treat..

[17] Press R.H., Zhang C., Chowdhary M., Prabhu R.S., Ferris M.J., Xu K.M., Olson J.J., Eaton B.R., Shu H.G., Curran W.J. (2018). Hemorrhagic and Cystic Brain Metastases Are Associated With an Increased Risk of Leptomeningeal Dissemination After Surgical Resection and Adjuvant Stereotactic Radiosurgery. Neurosurgery.

[18] Lamba N., Muskens I.S., DiRisio A.C., Meijer L., Briceno V., Edrees H., Aslam B., Minhas S., Verhoeff J.J.C., Kleynen C.E. (2017). Stereotactic radiosurgery versus whole-brain radiotherapy after intracranial metastasis resection: A systematic review and meta-analysis. Radiat. Oncol..

[19] Taggar A., MacKenzie J., Li H., Lau H., Lim G., Nordal R., Hudson A., Khan R., Spencer D., Voroney J.P. (2016). Survival was Significantly Better with Surgical/Medical/Radiation Co-interventions in a Single-Institution Practice Audit of Frameless Stereotactic Radiosurgery. Cureus.

[20] Soffietti R., Kocher M., Abacioglu U.M., Villa S., Fauchon F., Baumert B.G., Fariselli L., Tzuk-Shina T., Kortmann R.D., Carrie C. (2013). A European Organisation for Research and Treatment of Cancer phase III trial of adjuvant whole-brain radiotherapy versus observation in patients with one to three brain metastases from solid tumors after surgical resection or radiosurgery: Quality-of-life results. J. Clin. Oncol..

[21] Cummings M., Youn P., Bergsma D.P., Usuki K.Y., Walter K., Sharma M., Okunieff P., Schell M.C., Milano M.T. (2018). Single-Fraction Radiosurgery Using Conservative Doses for Brain Metastases: Durable Responses in Select Primaries with Limited Toxicity. Neurosurgery.

[22] Suzuki S., Inoue T., Ishido K. (2016). Factors influencing local tumor control after Gamma Knife radiosurgery for intracranial metastases from breast cancer. J. Clin. Neurosci..

[23] Wolf A., Kvint S., Chachoua A., Pavlick A., Wilson M., Donahue B., Golfinos J.G., Silverman J., Kondziolka D. (2018). Toward the complete control of brain metastases using surveillance screening and stereotactic radiosurgery. J. Neurosurg..

[24] Shen C.J., Rigamonti D., Redmond K.J., Kummerlowe M.N., Lim M., Kleinberg L.R. (2016). The strategy of repeat stereotactic radiosurgery without whole brain radiation treatment for new brain metastases: Outcomes and implications for follow-up monitoring. Pract. Radiat. Oncol.

[25] Kelly W.J., Shah N.J., Subramaniam D.S. (2018). Management of Brain Metastases in Epidermal Growth Factor Receptor Mutant Non-Small-Cell Lung Cancer. Front. Oncol..

[26] Magnuson W.J., Lester-Coll N.H., Wu A.J., Yang T.J., Lockney N.A., Gerber N.K., Beal K., Amini A., Patil T., Kavanagh B.D. (2017). Management of Brain Metastases in Tyrosine Kinase Inhibitor-Naive Epidermal Growth Factor Receptor-Mutant Non-Small-Cell Lung Cancer: A Retrospective Multi-Institutional Analysis. J. Clin. Oncol..

[27] Davies M.A., Saiag P., Robert C., Grob J.J., Flaherty K.T., Arance A., Chiarion-Sileni V., Thomas L., Lesimple T., Mortier L. (2017). Dabrafenib plus trametinib in patients with BRAF(V600)-mutant melanoma brain metastases (COMBI-MB): A multicenter, multicohort, open-label, phase 2 trial. Lancet Oncol..

[28] Lin N.U., Lee E.Q., Aoyama H., Barani I.J., Barboriak D.P., Baumert B.G., Bendszus M., Brown P.D., Camidge D.R., Chang S.M. (2015). Response assessment criteria for brain metastases: Proposal from the RANO group. Lancet Oncol..

[29] Gardner T.W., Lieth E., Khin S.A., Barber A.J., Bonsall D.J., Lesher T., Rice K., Brennan W.A. (1997). Astrocytes increase barrier properties and ZO-1 expression in retinal vascular endothelial cells. Investig. Ophthalmol. Vis. Sci..

[30] Kim J.H., Kim J.H., Yu Y.S., Kim D.H., Kim K.W. (2009). Recruitment of pericytes and astrocytes is closely related to the formation of tight junction in developing retinal vessels. J. Neurosci. Res..

[31] Balabanov R., Dore-Duffy P. (1998). Role of the CNS microvascular pericyte in the blood-brain barrier. J. Neurosci. Res..

[32] Bonkowski D., Katyshev V., Balabanov R.D., Borisov A., Dore-Duffy P. (2011). The CNS microvascular pericyte: Pericyte-astrocyte crosstalk in the regulation of tissue survival. Fluids Barriers CNS.

[33] Armulik A., Genové G., Mäe M., Nisancioglu M.H., Wallgard E., Niaudet C., He L., Norlin J., Lindblom P., Strittmatter K. (2010). Pericytes regulate the blood–brain barrier. Nature.

[34] Daneman R., Zhou L., Kebede A.A., Barres B.A. (2010). Pericytes are required for blood-brain barrier integrity during embryogenesis. Nature.

[35] Klemke M., Weschenfelder T., Konstandin M.H., Samstag Y. (2007). High affinity interaction of integrin alpha4beta1 (VLA-4) and vascular cell adhesion molecule 1 (VCAM-1) enhances migration of human melanoma cells across activated endothelial cell layers. J. Cell. Physiol..

[36] Wu K., Fukuda K., Xing F., Zhang Y., Sharma S., Liu Y., Chan M.D., Zhou X., Qasem S.A., Pochampally R. (2015). Roles of the cyclooxygenase 2 matrix metalloproteinase 1 pathway in brain metastasis of breast cancer. J. Biol. Chem..

[37] Pukrop T., Dehghani F., Chuang H.N., Lohaus R., Bayanga K., Heermann S., Regen T., Van Rossum D., Klemm F., Schulz M. (2010). Microglia promote colonization of brain tissue by breast cancer cells in a Wnt-dependent way. Glia.

[38] Custodio-Santos T., Videira M., Brito M.A. (2017). Brain metastasization of breast cancer. Biochim. Biophys. Acta Rev. Cancer.

[39] Li W., Graeber M.B. (2012). The molecular profile of microglia under the influence of glioma. Neuro-Oncology.

[40] Coniglio S.J., Segall J.E. (2013). Review: Molecular mechanism of microglia stimulated glioblastoma invasion. Matrix Biol..

[41] Gril B., Paranjape A.N., Woditschka S., Hua E., Dolan E.L., Hanson J., Wu X., Kloc W., Izycka-Swieszewska E., Duchnowska R. (2018). Reactive astrocytic S1P3 signaling modulates the blood–tumor barrier in brain metastases. Nat. Commun..

[42] Seike T., Fujita K., Yamakawa Y., Kido M.A., Takiguchi S., Teramoto N., Iguchi H., Noda M. (2011). Interaction between lung cancer cells and astrocytes via specific inflammatory cytokines in the microenvironment of brain metastasis. Clin. Exp. Metastasis.

[43] Hoshide R., Jandial R. (2017). The role of the neural niche in brain metastasis. Clin. Exp. Metastasis.

[44] Valiente M., Obenauf A.C., Jin X., Chen Q., Zhang X.H., Lee D.J., Chaft J.E., Kris M.G., Huse J.T., Brogi E. (2014). Serpins promote cancer cell survival and vascular co-option in brain metastasis. Cell.

[45] Orozco J.I.J., Knijnenburg T.A., Manughian-Peter A.O., Salomon M.P., Barkhoudarian G., Jalas J.R., Wilmott J.S., Hothi P., Wang X., Takasumi Y. (2018). Epigenetic profiling for the molecular classification of metastatic brain tumors. Nat. Commun..

[46] Saunus J.M., McCart Reed A.E., Lim Z.L., Lakhani S.R. (2017). Breast Cancer Brain Metastases: Clonal Evolution in Clinical Context. Int. J. Mol. Sci..

[47] Wronski M., Arbit E. (2000). Surgical treatment of brain metastases from melanoma: A retrospective study of 91 patients. J. Neurosurg..

[48] Berghoff A.S., Rajky O., Winkler F., Bartsch R., Furtner J., Hainfellner J.A., Goodman S.L., Weller M., Schittenhelm J., Preusser M. (2013). Invasion patterns in brain metastases of solid cancers. Neuro-Oncology.

[49] Pekmezci M., Perry A. (2013). Neuropathology of brain metastases. Surg. Neurol. Int..

[50] Fidler I.J., Balasubramanian K., Lin Q., Kim S.W., Kim S.J. (2010). The brain microenvironment and metastasis. Mol. Cells.

[51] Gril B., Palmieri D., Qian Y., Anwar T., Liewehr D.J., Steinberg S.M., Andreu Z., Masana D., Fernandez P., Steeg P.S. (2013). Pazopanib inhibits the activation of PDGFR beta-expressing astrocytes in the brain metastatic microenvironment of breast cancer cells. Am. J. Pathol..

[52] Neman J., Choy C., Kowolik C.M., Anderson A., Duenas V.J., Waliany S., Chen B.T., Chen M.Y., Jandial R. (2013). Co-evolution of breast-to-brain metastasis and neural progenitor cells. Clin. Exp. Metastasis.

[53] Neman J., Termini J., Wilczynski S., Vaidehi N., Choy C., Kowolik C.M., Li H., Hambrecht A.C., Roberts E., Jandial R. (2014). Human breast cancer metastases to the brain display GABAergic properties in the neural niche. Proc. Natl. Acad. Sci. USA.

[54] Sevenich L., Bowman R.L., Mason S.D., Quail D.F., Rapaport F., Elie B.T., Brogi E., Brastianos P.K., Hahn W.C., Holsinger L.J. (2014). Analysis of tumor- and stroma-supplied proteolytic networks reveals a brain-metastasis-promoting role for cathepsin S. Nat. Cell Biol..

[55] Da Silva L., Simpson P.T., Smart C.E., Cocciardi S., Waddell N., Lane A., Morrison B.J., Vargas A., Healey S., Beesley J. (2010). HER3 and downstream pathways are involved in colonization of brain metastases from breast cancer. Breast Cancer Res..

[56] Preusser M., Streubel B., Berghoff A.S., Hainfellner J.A., von Deimling A., Widhalm G., Dieckmann K., Wohrer A., Hackl M., Zielinski C. (2014). Amplification and overexpression of CMET is a common event in brain metastases of non-small cell lung cancer. Histopathology.

[57] Saunus J.M., Quinn M.C., Patch A.M., Pearson J.V., Bailey P.J., Nones K., McCart Reed A.E., Miller D., Wilson P.J., Al-Ejeh F. (2015). Integrated genomic and transcriptomic analysis of human brain metastases identifies alterations of potential clinical significance. J. Pathol..

[58] Sun M., Behrens C., Feng L., Ozburn N., Tang X., Yin G., Komaki R., Varella-Garcia M., Hong W.K., Aldape K.D. (2009). HER family receptor abnormalities in lung cancer brain metastases and corresponding primary tumors. Clin. Cancer Res..

[59] Wikman H., Lamszus K., Detels N., Uslar L., Wrage M., Benner C., Hohensee I., Ylstra B., Eylmann K., Zapatka M. (2012). Relevance of PTEN loss in brain metastasis formation in breast cancer patients. Breast Cancer Res..

[60] Brastianos P.K., Carter S.L., Santagata S., Cahill D.P., Taylor-Weiner A., Jones R.T., Van Allen E.M., Lawrence M.S., Horowitz P.M., Cibulskis K. (2015). Genomic Characterization of Brain Metastases Reveals Branched Evolution and Potential Therapeutic Targets. Cancer Discov..

[61] Lee J.Y., Park K., Lim S.H., Kim H.S., Yoo K.H., Jung K.S., Song H.-N., Hong M., Do I.-G., Ahn T. (2015). Mutational profiling of brain metastasis from breast cancer: Matched pair analysis of targeted sequencing between brain metastasis and primary breast cancer. Oncotarget.

[62] Zhang L., Zhang S., Yao J., Lowery F.J., Zhang Q., Huang W.C., Li P., Li M., Wang X., Zhang C. (2015). Microenvironment-induced PTEN loss by exosomal microRNA primes brain metastasis outgrowth. Nature.

[63] Aurilio G., Disalvatore D., Pruneri G., Bagnardi V., Viale G., Curigliano G., Adamoli L., Munzone E., Sciandivasci A., De Vita F. (2014). A meta-analysis of estrogen receptor, progesterone receptor and human epidermal growth factor receptor 2 discordance between primary breast cancer and metastases. Eur. J. Cancer.

[64] Ding L., Ellis M.J., Li S., Larson D.E., Chen K., Wallis J.W., Harris C.C., McLellan M.D., Fulton R.S., Fulton L.L. (2010). Genome remodelling in a basal-like breast cancer metastasis and xenograft. Nature.

[65] Poznak C.V., Somerfield M.R., Bast R.C., Cristofanilli M., Goetz M.P., Gonzalez-Angulo A.M., Hicks D.G., Hill E.G., Liu M.C., Lucas W. (2015). Use of Biomarkers to Guide Decisions on Systemic Therapy for Women with Metastatic Breast Cancer: American Society of Clinical Oncology Clinical Practice Guideline. J. Clin. Oncol..

[66] Berghoff A.S., Ilhan-Mutlu A., Wohrer A., Hackl M., Widhalm G., Hainfellner J.A., Dieckmann K., Melchardt T., Dome B., Heinzl H. (2014). Prognostic significance of Ki67 proliferation index, HIF1 alpha index and microvascular density in patients with non-small cell lung cancer brain metastases. Strahlenther. Onkol..

[67] Vaupel P., Mayer A. (2005). Hypoxia and anemia: Effects on tumor biology and treatment resistance. Transfus. Clin. Biol..

[68] Sundahl N., Duprez F., Ost P., De Neve W., Mareel M. (2018). Effects of radiation on the metastatic process. Mol. Med. (Cambridge, Mass.).

[69] Kumar P. (2000). Tumor hypoxia and anemia: Impact on the efficacy of radiation therapy. Semin. Hematol..

[70] Clarke R.H., Moosa S., Anzivino M., Wang Y., Floyd D.H., Purow B.W., Lee K.S. (2014). Sustained radiosensitization of hypoxic glioma cells after oxygen pretreatment in an animal model of glioblastoma and in vitro models of tumor hypoxia. PLoS ONE.

[71] De Bacco F., Luraghi P., Medico E., Reato G., Girolami F., Perera T., Gabriele P., Comoglio P.M., Boccaccio C. (2011). Induction of MET by ionizing radiation and its role in radioresistance and invasive growth of cancer. J. Natl. Cancer Inst..

[72] Bhardwaj V., Zhan Y., Cortez M.A., Ang K.K., Molkentine D., Munshi A., Raju U., Komaki R., Heymach J.V., Welsh J. (2012). C-Met inhibitor MK-8003 radiosensitizes c-Met-expressing non-small-cell lung cancer cells with radiation-induced c-Met-expression. J. Thorac. Oncol..

[73] Wang P., Xiao P., Ye Y., Liu P., Han L., Dong L., She C., Yu J. (2017). Rapid response of brain metastasis to crizotinib in a patient with KLC1-ALK fusion and MET gene amplification positive non-small cell lung cancer: A case report. Cancer Biol. Med..

[74] Negrier S., Moriceau G., Attignon V., Haddad V., Pissaloux D., Guerin N., Carrie C. (2018). Activity of cabozantinib in radioresistant brain metastases from renal cell carcinoma: Two case reports. J. Med. Case Rep..

[75] Momeny M., Saunus J.M., Marturana F., McCart Reed A.E., Black D., Sala G., Iacobelli S., Holland J.D., Yu D., Da Silva L. (2015). Heregulin-HER3-HER2 signaling promotes matrix metalloproteinase-dependent blood-brain-barrier transendothelial migration of human breast cancer cell lines. Oncotarget.

[76] Kienast Y., von Baumgarten L., Fuhrmann M., Klinkert W.E., Goldbrunner R., Herms J., Winkler F. (2010). Real-time imaging reveals the single steps of brain metastasis formation. Nat. Med..

[77] Carmeliet P., Jain R.K. (2011). Principles and mechanisms of vessel normalization for cancer and other angiogenic diseases. Nat. Rev. Drug Discov..

[78] Monsky W.L., Mouta Carreira C., Tsuzuki Y., Gohongi T., Fukumura D., Jain R.K. (2002). Role of host microenvironment in angiogenesis and microvascular functions in breast cancer xenografts: MFP vs cranial tumors. Clin. Cancer Res..

[79] Jain R.K., Martin J.D., Stylianopoulos T. (2014). The role of mechanical forces in tumor growth and therapy. Annu. Rev. Biomed. Eng..

[80] Munson J.M., Shieh A.C. (2014). Interstitial fluid flow in cancer: Implications for disease progression and treatment. Cancer Manag. Res..

[81] Sarkiss C.A., Germano I.M. (2019). Machine Learning in Neuro-Oncology: Can Data Analysis from 5,346 Patients Change Decision Making Paradigms?. World Neurosurg..

[82] Lotan E., Jain R., Razavian N., Fatterpekar G.M., Lui Y.W. (2019). State of the Art: Machine Learning Applications in Glioma Imaging. AJR. Am. J. roentgenol..

[83] Ainsworth N.L., McLean M.A., McIntyre D.J.O., Honess D.J., Brown A.M., Harden S.V., Griffiths J.R. (2017). Quantitative and textural analysis of magnetization transfer and diffusion images in the early detection of brain metastases. Magn. Reson. Med..

[84] Yin G., Li C., Chen H., Luo Y., Orlandini L.C., Wang P., Lang J. (2017). Predicting brain metastases for non-small cell lung cancer based on magnetic resonance imaging. Clin. Exp. Metastasis.

[85] Niessner H., Schmitz J., Tabatabai G., Schmid A.M., Calaminus C., Sinnberg T., Weide B., Eigentler T.K., Garbe C., Schittek B. (2016). PI3K Pathway Inhibition Achieves Potent Antitumor Activity in Melanoma Brain Metastases *In Vitro* and *In Vivo*. Clin. Cancer Res..

[86] Ni J., Ramkissoon S.H., Xie S., Goel S., Stover D.G., Guo H., Luu V., Marco E., Ramkissoon L.A., Kang Y.J. (2016). Combination inhibition of PI3K and mTORC1 yields durable remissions in mice bearing orthotopic patient-derived xenografts of HER2-positive breast cancer brain metastases. Nat. Med..

[87] Kodack D.P., Askoxylakis V., Ferraro G.B., Sheng Q., Badeaux M., Goel S., Qi X., Shankaraiah R., Cao Z.A., Ramjiawan R.R. (2017). The brain microenvironment mediates resistance in luminal breast cancer to PI3K inhibition through HER3 activation. Sci. Transl. Med..

[88] André F., Kaufman B., Juric D., Ciruelos E., Iwata H., Mayer I.A., Conte P., Rugo H.S., Loibl S., Rubovszky G. (2016). A phase III study of alpelisib and fulvestrant in men and postmenopausal women with hormone receptor-positive (HR+), human epidermal growth factor receptor 2-negative (HER2–) advanced breast cancer (BC) progressing on or after aromatase inhibitor (AI) therapy (SOLAR-1). Ann. Oncol..

[89] De Gooijer M.C., Zhang P., Buil L.C.M., Çitirikkaya C.H., Thota N., Beijnen J.H., van Tellingen O. (2018). Buparlisib is a brain penetrable pan-PI3K inhibitor. Sci. Rep..

[90] Koul D., Fu J., Shen R., LaFortune T.A., Wang S., Tiao N., Kim Y.W., Liu J.L., Ramnarian D., Yuan Y. (2012). Antitumor activity of NVP-BKM120—A selective pan class I PI3 kinase inhibitor showed differential forms of cell death based on p53 status of glioma cells. Clin. Cancer Res..

[91] Rodon J., Brana I., Siu L.L., De Jonge M.J., Homji N., Mills D., Di Tomaso E., Sarr C., Trandafir L., Massacesi C. (2014). Phase I dose-escalation and -expansion study of buparlisib (BKM120), an oral pan-Class I PI3K inhibitor, in patients with advanced solid tumors. Investig. New Drugs.

[92] Baselga J., Im S.-A., Iwata H., Cortés J., De Laurentiis M., Jiang Z., Arteaga C.L., Jonat W., Clemons M., Ito Y. (2017). Buparlisib plus fulvestrant versus placebo plus fulvestrant in postmenopausal, hormone receptor-positive, HER2-negative, advanced breast cancer (BELLE-2): A randomized, double-blind, placebo-controlled, phase 3 trial. Lancet Oncol..

[93] Di Leo A., Johnston S., Lee K.S., Ciruelos E., Lønning P.E., Janni W., O’Regan R., Mouret-Reynier M.-A., Kalev D., Egle D. (2018). Buparlisib plus fulvestrant in postmenopausal women with hormone-receptor-positive, HER2-negative, advanced breast cancer progressing on or after mTOR inhibition (BELLE-3): A randomized, double-blind, placebo-controlled, phase 3 trial. Lancet Oncol..

[94] Mendell J., Freeman D.J., Feng W., Hettmann T., Schneider M., Blum S., Ruhe J., Bange J., Nakamaru K., Chen S. (2015). Clinical Translation and Validation of a Predictive Biomarker for Patritumab, an Anti-human Epidermal Growth Factor Receptor 3 (HER3) Monoclonal Antibody, in Patients With Advanced Non-small Cell Lung Cancer. EBioMedicine.

[95] Mirschberger C., Schiller C.B., Schräml M., Dimoudis N., Friess T., Gerdes C.A., Reiff U., Lifke V., Hoelzlwimmer G., Kolm I. (2013). RG7116, a Therapeutic Antibody That Binds the Inactive HER3 Receptor and Is Optimized for Immune Effector Activation. Cancer Res..

[96] Berghoff A.S., Magerle M., Ilhan-Mutlu A., Dinhof C., Widhalm G., Dieckman K., Marosi C., Wohrer A., Hackl M., Zochbauer-Muller S. (2013). Frequent overexpression of ErbB—Receptor family members in brain metastases of non-small cell lung cancer patients. APMIS.

[97] Berghoff A.S., Bago-Horvath Z., Ilhan-Mutlu A., Magerle M., Dieckmann K., Marosi C., Birner P., Widhalm G., Steger G.G., Zielinski C.C. (2012). Brain-only metastatic breast cancer is a distinct clinical entity characterized by favourable median overall survival time and a high rate of long-term survivors. Br. J. Cancer.

[98] Xia W., Petricoin E.F., Zhao S., Liu L., Osada T., Cheng Q., Wulfkuhle J.D., Gwin W.R., Yang X., Gallagher R.I. (2013). An heregulin-EGFR-HER3 autocrine signaling axis can mediate acquired lapatinib resistance in HER2+ breast cancer models. Breast Cancer Res..

[99] Sergina N.V., Rausch M., Wang D., Blair J., Hann B., Shokat K.M., Moasser M.M. (2007). Escape from HER-family tyrosine kinase inhibitor therapy by the kinase-inactive HER3. Nature.

[100] Tolaney S.M., Boucher Y., Duda D.G., Martin J.D., Seano G., Ancukiewicz M., Barry W.T., Goel S., Lahdenrata J., Isakoff S.J. (2015). Role of vascular density and normalization in response to neoadjuvant bevacizumab and chemotherapy in breast cancer patients. Proc. Natl. Acad. Sci. USA.

[101] US National Institutes of Health. www.clinicaltrials.gov.

[102] Yang J., Yan J., Liu B. (2018). Targeting VEGF/VEGFR to Modulate Antitumor Immunity. Front. Immunol..

[103] Diossy M., Reiniger L., Sztupinszki Z., Krzystanek M., Timms K.M., Neff C., Solimeno C., Pruss D., Eklund A.C., Tóth E. (2018). Breast cancer brain metastases show increased levels of genomic aberration-based homologous recombination deficiency scores relative to their corresponding primary tumors. Ann. Oncol..

[104] McMullin R.P., Wittner B.S., Yang C., Denton-Schneider B.R., Hicks D., Singavarapu R., Moulis S., Lee J., Akbari M.R., Narod S.A. (2014). A BRCA1 deficient-like signature is enriched in breast cancer brain metastases and predicts DNA damage-induced poly (ADP-ribose) polymerase inhibitor sensitivity. Breast Cancer Res. BCR.

[105] Mehta M.P., Wang D., Wang F., Kleinberg L., Brade A., Robins H.I., Turaka A., Leahy T., Medina D., Xiong H. (2015). Veliparib in combination with whole brain radiation therapy in patients with brain metastases: Results of a phase 1 study. J. Neurooncol..

[106] Chabot P., Hsia T.C., Ryu J.S., Gorbunova V., Belda-Iniesta C., Ball D., Kio E., Mehta M., Papp K., Qin Q. (2017). Veliparib in combination with whole-brain radiation therapy for patients with brain metastases from non-small cell lung cancer: Results of a randomized, global, placebo-controlled study. J. Neurooncol..

[107] Anders C., Deal A.M., Abramson V., Liu M.C., Storniolo A.M., Carpenter J.T., Puhalla S., Nanda R., Melhem-Bertrandt A., Lin N.U. (2014). TBCRC 018: Phase II study of iniparib in combination with irinotecan to treat progressive triple negative breast cancer brain metastases. Breast Cancer Res. Treat..

[108] Licht S., Cao H., Li Z., Zhang J., Liu F., Brittain S., Shen J., Zhang B., Hopke J., Newcombe R. (2011). Abstract A226: Mechanism of action of iniparib: Stimulation of reactive oxygen species (ROS) production in an iniparib-sensitive breast cancer cell line. Mol. Cancer Ther..

[109] Triguero D., Buciak J.B., Yang J., Pardridge W.M. (1989). Blood-brain barrier transport of cationized immunoglobulin G: Enhanced delivery compared to native protein. Proc. Natl. Acad. Sci. USA.

[110] Hervé F., Ghinea N., Scherrmann J.-M. (2008). CNS Delivery Via Adsorptive Transcytosis. AAPS J..

[111] Qin Y., Fan W., Chen H., Yao N., Tang W., Tang J., Yuan W., Kuai R., Zhang Z., Wu Y. (2010). In vitro and in vivo investigation of glucose-mediated brain-targeting liposomes. J. Drug Target..

[112] Jiang Y.Z., Yu K.D., Bao J., Peng W.T., Shao Z.M. (2014). Favorable Prognostic Impact in Loss of TP53 and PIK3CA Mutations after Neoadjuvant Chemotherapy in Breast Cancer. Cancer Res..

[113] Dickens D., Webb S.D., Antonyuk S., Giannoudis A., Owen A., Radisch S., Hasnain S.S., Pirmohamed M. (2013). Transport of gabapentin by LAT1 (SLC7A5). Biochem. Pharmacol..

[114] Gomes P., Soares-da-Silva P. (1999). L-DOPA transport properties in an immortalized cell line of rat capillary cerebral endothelial cells, RBE 4. Brain Res..

[115] Van Bree J.B., Audus K.L., Borchardt R.T. (1988). Carrier-mediated transport of baclofen across monolayers of bovine brain endothelial cells in primary culture. Pharm. Res..

[116] Yu Y.J., Zhang Y., Kenrick M., Hoyte K., Luk W., Lu Y., Atwal J., Elliott J.M., Prabhu S., Watts R.J. (2011). Boosting Brain Uptake of a Therapeutic Antibody by Reducing Its Affinity for a Transcytosis Target. Sci. Transl. Med..

[117] Bien-Ly N., Yu Y.J., Bumbaca D., Elstrott J., Boswell C.A., Zhang Y., Luk W., Lu Y., Dennis M.S., Weimer R.M. (2014). Transferrin receptor (TfR) trafficking determines brain uptake of TfR antibody affinity variants. J. Exp. Med..

[118] Régina A., Demeule M., Ché C., Lavallée I., Poirier J., Gabathuler R., Béliveau R., Castaigne J.P. (2008). Antitumor activity of ANG1005, a conjugate between paclitaxel and the new brain delivery vector Angiopep-2. Br. J. Pharmacol..

[119] Drappatz J., Brenner A., Wong E.T., Eichler A., Schiff D., Groves M.D., Mikkelsen T., Rosenfeld S., Sarantopoulos J., Meyers C.A. (2013). Phase I study of GRN1005 in recurrent malignant glioma. Clin. Cancer Res..

[120] Kurzrock R., Gabrail N., Chandhasin C., Moulder S., Smith C., Brenner A., Sankhala K., Mita A., Elian K., Bouchard D. (2012). Safety, pharmacokinetics, and activity of GRN1005, a novel conjugate of angiopep-2, a peptide facilitating brain penetration, and paclitaxel, in patients with advanced solid tumors. Mol. Cancer Ther..

[121] Tang S.C., Kumthekar P., Brenner A.J., Kesari S., Piccioni D., Anders C.K., Carillo J.A., Chalasani P., Kabos P., Puhalla S.L. (2016). ANG1005, a novel peptide-paclitaxel conjugate crosses the BBB and shows activity in patients with recurrent CNS metastasis from breast cancer, results from a phase II clinical study. Ann. Oncol..

[122] Lin N.U., Gabrail N.Y., Sarantopoulos J., Schwartzberg L.S., Kesari S., Bates S.E., Anders C.K., Elias A.D., Castaigne J.-P., Iordanova V. (2014). Evaluation of CNS and peripheral antitumor activity of ANG1005 in patients with brain metastases from breast tumors and other advanced solid tumors. J. Clin. Oncol..

[123] De Gooijer M.C., de Vries N.A., Buckle T., Buil L.C.M., Beijnen J.H., Boogerd W., van Tellingen O. (2018). Improved Brain Penetration and Antitumor Efficacy of Temozolomide by Inhibition of ABCB1 and ABCG2. Neoplasia (New York, N.Y.).

[124] Kuntner C., Bankstahl J.P., Bankstahl M., Stanek J., Wanek T., Stundner G., Karch R., Brauner R., Meier M., Ding X. (2010). Dose-response assessment of tariquidar and elacridar and regional quantification of P-glycoprotein inhibition at the rat blood-brain barrier using (R)-[(11)C]verapamil PET. Eur. J. Nucl. Med. Mol. Imaging.

[125] Jablonski M.R., Markandaiah S.S., Jacob D., Meng N.J., Li K., Gennaro V., Lepore A.C., Trotti D., Pasinelli P. (2014). Inhibiting drug efflux transporters improves efficacy of ALS therapeutics. Ann. Clin. Transl. Neurol..

[126] Fracasso P.M., Brady M.F., Moore D.H., Walker J.L., Rose P.G., Letvak L., Grogan T.M., McGuire W.P. (2001). Phase II Study of Paclitaxel and Valspodar (PSC 833) in Refractory Ovarian Carcinoma: A Gynecologic Oncology Group Study. J. Clin. Oncol..

[127] Seiden M.V., Swenerton K.D., Matulonis U., Campos S., Rose P., Batist G., Ette E., Garg V., Fuller A., Harding M.W. (2002). A Phase II Study of the MDR Inhibitor Biricodar (INCEL, VX-710) and Paclitaxel in Women with Advanced Ovarian Cancer Refractory to Paclitaxel Therapy. Gynecol. Oncol..

[128] Pusztai L., Wagner P., Ibrahim N., Rivera E., Theriault R., Booser D., Symmans F.W., Wong F., Blumenschein G., Fleming D.R. (2005). Phase II study of tariquidar, a selective P-glycoprotein inhibitor, in patients with chemotherapy-resistant, advanced breast carcinoma. Cancer.

[129] Lhomme C., Joly F., Walker J.L., Lissoni A.A., Nicoletto M.O., Manikhas G.M., Baekelandt M.M., Gordon A.N., Fracasso P.M., Mietlowski W.L. (2008). Phase III study of valspodar (PSC 833) combined with paclitaxel and carboplatin compared with paclitaxel and carboplatin alone in patients with stage IV or suboptimally debulked stage III epithelial ovarian cancer or primary peritoneal cancer. J. Clin. Oncol..

[130] Fox E., Widemann B.C., Pastakia D., Chen C.C., Yang S.X., Cole D., Balis F.M. (2015). Pharmacokinetic and pharmacodynamic study of tariquidar (XR9576), a P-glycoprotein inhibitor, in combination with doxorubicin, vinorelbine, or docetaxel in children and adolescents with refractory solid tumors. Cancer Chemother. Pharmacol..

[131] Kelly R.J., Draper D., Chen C.C., Robey R.W., Figg W.D., Piekarz R.L., Chen X., Gardner E.R., Balis F.M., Venkatesan A.M. (2011). A Pharmacodynamic Study of Docetaxel in Combination with the P-glycoprotein Antagonist Tariquidar (XR9576) in Patients with Lung, Ovarian, and Cervical Cancer. Clin. Cancer Res..

[132] Hernangómez M., Carrillo-Salinas F.J., Mecha M., Correa F., Mestre L., Loría F., Feliú A., Docagne F., Guaza C. (2014). Brain innate immunity in the regulation of neuroinflammation: Therapeutic strategies by modulating CD200-CD200R interaction involve the cannabinoid system. Curr. Pharm. Des..

[133] Medzhitov R., Janeway C.A. (2002). Decoding the patterns of self and nonself by the innate immune system. Science.

[134] Elward K., Gasque P. (2003). “Eat me” and “don’t eat me” signals govern the innate immune response and tissue repair in the CNS: Emphasis on the critical role of the complement system. Mol. Immunol..

[135] Ransohoff R.M., Brown M.A. (2012). Innate immunity in the central nervous system. J. Clin. Investig..

[136] Gasque P., Dean Y.D., McGreal E.P., Vanbeek J., Morgan B.P. (2000). Complement components of the innate immune system in health and disease in the CNS. Immunopharmacology.

[137] Magnus T., Chan A., Linker R.A., Toyka K.V., Gold R. (2002). Astrocytes are less efficient in the removal of apoptotic lymphocytes than microglia cells: Implications for the role of glial cells in the inflamed central nervous system. J. Neuropathol. Exp. Neurol..

[138] Louveau A., Smirnov I., Keyes T.J., Eccles J.D., Rouhani S.J., Peske J.D., Derecki N.C., Castle D., Mandell J.W., Lee K.S. (2015). Structural and functional features of central nervous system lymphatic vessels. Nature.

[139] Bartholomaus I., Kawakami N., Odoardi F., Schlager C., Miljkovic D., Ellwart J.W., Klinkert W.E., Flugel-Koch C., Issekutz T.B., Wekerle H. (2009). Effector T cell interactions with meningeal vascular structures in nascent autoimmune CNS lesions. Nature.

[140] Kawakami N., Nagerl U.V., Odoardi F., Bonhoeffer T., Wekerle H., Flugel A. (2005). Live imaging of effector cell trafficking and autoantigen recognition within the unfolding autoimmune encephalomyelitis lesion. J. Exp. Med..

[141] Cserr H.F., Harling-Berg C.J., Knopf P.M. (1992). Drainage of brain extracellular fluid into blood and deep cervical lymph and its immunological significance. Brain Pathol..

[142] Kida S., Pantazis A., Weller R.O. (1993). CSF drains directly from the subarachnoid space into nasal lymphatics in the rat. Anatomy, histology and immunological significance. Neuropathol. Appl. Neurobiol..

[143] Iorgulescu J.B., Harary M., Zogg C.K., Ligon K.L., Reardon D.A., Hodi F.S., Aizer A.A., Smith T.R. (2018). Improved Risk-Adjusted Survival for Melanoma Brain Metastases in the Era of Checkpoint Blockade Immunotherapies: Results from a National Cohort. Cancer Immunol. Res..

[144] Schartz N.E., Farges C., Madelaine I., Bruzzoni H., Calvo F., Hoos A., Lebbe C. (2010). Complete regression of a previously untreated melanoma brain metastasis with ipilimumab. Melanoma Res..

[145] Margolin K., Ernstoff M.S., Hamid O., Lawrence D., McDermott D., Puzanov I., Wolchok J.D., Clark J.I., Sznol M., Logan T.F. (2012). Ipilimumab in patients with melanoma and brain metastases: An open-label, phase 2 trial. Lancet Oncol..

[146] Weber J.S., Amin A., Minor D., Siegel J., Berman D., O’Day S.J. (2011). Safety and clinical activity of ipilimumab in melanoma patients with brain metastases: Retrospective analysis of data from a phase 2 trial. Melanoma Res..

[147] Goldberg S.B., Gettinger S.N., Mahajan A., Chiang A.C., Herbst R.S., Sznol M., Tsiouris A.J., Cohen J., Vortmeyer A., Jilaveanu L. (2016). Pembrolizumab for patients with melanoma or non-small-cell lung cancer and untreated brain metastases: Early analysis of a non-randomized, open-label, phase 2 trial. Lancet Oncol..

[148] Kamath S.D., Kumthekar P.U. (2018). Immune Checkpoint Inhibitors for the Treatment of Central Nervous System (CNS) Metastatic Disease. Front. Oncol..

[149] Tawbi H.A., Forsyth P.A., Algazi A., Hamid O., Hodi F.S., Moschos S.J., Khushalani N.I., Lewis K., Lao C.D., Postow M.A. (2018). Combined Nivolumab and Ipilimumab in Melanoma Metastatic to the Brain. N. Eng. J. Med..

[150] Taggart D., Andreou T., Scott K.J., Williams J., Rippaus N., Brownlie R.J., Ilett E.J., Salmond R.J., Melcher A., Lorger M. (2018). Anti–PD-1/anti–CTLA-4 efficacy in melanoma brain metastases depends on extracranial disease and augmentation of CD8+ T cell trafficking. Proc. Natl. Acad. Sci. USA.

[151] Murphy B., Walker J., Bassale S., Monaco D., Jaboin J., Ciporen J., Taylor M., Dai Kubicky C. (2018). Concurrent Radiosurgery and Immune Checkpoint Inhibition: Improving Regional Intracranial Control for Patients with Metastatic Melanoma. Am. J. Clin. Oncol..

[152] Fleming V., Hu X., Weber R., Nagibin V., Groth C., Altevogt P., Utikal J., Umansky V. (2018). Targeting Myeloid-Derived Suppressor Cells to Bypass Tumor-Induced Immunosuppression. Front. Immunol..

[153] Feng P.H., Chen K.Y., Huang Y.C., Luo C.S., Wu S.M., Chen T.T., Lee C.N., Yeh C.T., Chuang H.C., Han C.L. (2018). Bevacizumab Reduces S100A9-Positive MDSCs Linked to Intracranial Control in Patients with EGFR-Mutant Lung Adenocarcinoma. J. Thorac. Oncol..

[154] Du Four S., Maenhout S.K., De Pierre K., Renmans D., Niclou S.P., Thielemans K., Neyns B., Aerts J.L. (2015). Axitinib increases the infiltration of immune cells and reduces the suppressive capacity of monocytic MDSCs in an intracranial mouse melanoma model. Oncoimmunology.

[155] Bright R.K., Bright J.D., Byrne J.A. (2014). Overexpressed oncogenic tumor-self antigens. Hum. Vaccin. Immunother..

[156] Du W., Seah I., Bougazzoul O., Choi G., Meeth K., Bosenberg M.W., Wakimoto H., Fisher D., Shah K. (2017). Stem cell-released oncolytic herpes simplex virus has therapeutic efficacy in brain metastatic melanomas. Proc. Natl. Acad. Sci. USA.

[157] Arbour K.C., Mezquita L., Long N., Rizvi H., Auclin E., Ni A., Martinez-Bernal G., Ferrara R., Lai W.V., Hendriks L.E.L. (2018). Impact of Baseline Steroids on Efficacy of Programmed Cell Death-1 and Programmed Death-Ligand 1 Blockade in Patients with Non-Small-Cell Lung Cancer. J. Clin. Oncol..

[158] Caponnetto S., Draghi A., Borch T.H., Nuti M., Cortesi E., Svane I.M., Donia M. (2018). Cancer immunotherapy in patients with brain metastases. Cancer Immunol. Immunother..

[159] Boogerd W., Vos V.W., Hart A.A., Baris G. (1993). Brain metastases in breast cancer; natural history, prognostic factors and outcome. J. Neurooncol..

[160] Rosner D., Nemoto T., Lane W.W. (1986). Chemotherapy induces regression of brain metastases in breast carcinoma. Cancer.

[161] Woditschka S., Evans L., Duchnowska R., Reed L.T., Palmieri D., Qian Y., Badve S., Sledge G., Gril B., Aladjem M.I. (2014). DNA double-strand break repair genes and oxidative damage in brain metastasis of breast cancer. J. Natl. Cancer Inst..

[162] Grimm S.A. (2012). Treatment of brain metastases: Chemotherapy. Curr. Oncol. Rep..

[163] Owens D.E., Peppas N.A. (2006). Opsonization, biodistribution, and pharmacokinetics of polymeric nanoparticles. Int. J. Pharm..

[164] Nance E., Timbie K., Miller G.W., Song J., Louttit C., Klibanov A.L., Shih T.-Y., Swaminathan G., Tamargo R.J., Woodworth G.F. (2014). Noninvasive delivery of stealth, brain-penetrating nanoparticles across the blood-brain barrier using MRI-guided focused ultrasound. J. Control. Release Off. J. Control. Release Soc..

[165] Mattheolabakis G., Wong C.C., Sun Y., Amella C.A., Richards R., Constantinides P.P., Rigas B. (2014). Pegylation Improves the Pharmacokinetics and Bioavailability of Small-Molecule Drugs Hydrolyzable by Esterases: A Study of Phospho-Ibuprofen. J. Pharmacol. Exp. Ther..

[166] Howard C.B., Fletcher N., Houston Z.H., Fuchs A.V., Boase N.R., Simpson J.D., Raftery L.J., Ruder T., Jones M.L., de Bakker C.J. (2016). Overcoming Instability of Antibody-Nanomaterial Conjugates: Next Generation Targeted Nanomedicines Using Bispecific Antibodies. Adv. Healthc. Mater..

[167] Li J., Cai P., Shalviri A., Henderson J.T., He C., Foltz W.D., Prasad P., Brodersen P.M., Chen Y., DaCosta R. (2014). A multifunctional polymeric nanotheranostic system delivers doxorubicin and imaging agents across the blood-brain barrier targeting brain metastases of breast cancer. ACS Nano.

[168] Mittapalli R.K., Liu X., Adkins C.E., Nounou M.I., Bohn K.A., Terrell T.B., Qhattal H.S., Geldenhuys W.J., Palmieri D., Steeg P.S. (2013). Paclitaxel-hyaluronic nanoconjugates prolong overall survival in a preclinical brain metastases of breast cancer model. Mol. Cancer Ther..

[169] Hamilton A.M., Aidoudi-Ahmed S., Sharma S., Kotamraju V.R., Foster P.J., Sugahara K.N., Ruoslahti E., Rutt B.K. (2015). Nanoparticles coated with the tumor-penetrating peptide iRGD reduce experimental breast cancer metastasis in the brain. J. Mol. Med. (Berl.).

[170] Patil R., Ljubimov A.V., Gangalum P.R., Ding H., Portilla-Arias J., Wagner S., Inoue S., Konda B., Rekechenetskiy A., Chesnokova A. (2015). MRI virtual biopsy and treatment of brain metastatic tumors with targeted nanobioconjugates: Nanoclinic in the brain. ACS Nano.

[171] Bobo D., Robinson K.J., Islam J., Thurecht K.J., Corrie S.R. (2016). Nanoparticle-Based Medicines: A Review of FDA-Approved Materials and Clinical Trials to Date. Pharm. Res..

[172] Fan C.H., Cheng Y.H., Ting C.Y., Ho Y.J., Hsu P.H., Liu H.L., Yeh C.K. (2016). Ultrasound/Magnetic Targeting with SPIO-DOX-Microbubble Complex for Image-Guided Drug Delivery in Brain Tumors. Theranostics.

[173] Leinenga G., Gotz J. (2015). Scanning ultrasound removes amyloid-beta and restores memory in an Alzheimer’s disease mouse model. Sci. Transl. Med..

[174] Liu H.L., Fan C.H., Ting C.Y., Yeh C.K. (2014). Combining microbubbles and ultrasound for drug delivery to brain tumors: Current progress and overview. Theranostics.

[175] Chen H., Konofagou E.E. (2014). The size of blood-brain barrier opening induced by focused ultrasound is dictated by the acoustic pressure. J. Cereb. Blood Flow Metab..

[176] Deng C.X. (2010). Targeted drug delivery across the blood-brain barrier using ultrasound technique. Ther. Deliv..

[177] Kinoshita M., McDannold N., Jolesz F.A., Hynynen K. (2006). Noninvasive localized delivery of Herceptin to the mouse brain by MRI-guided focused ultrasound-induced blood-brain barrier disruption. Proc. Natl. Acad. Sci USA.

[178] Shen Y., Pi Z., Yan F., Yeh C.K., Zeng X., Diao X., Hu Y., Chen S., Chen X., Zheng H. (2017). Enhanced delivery of paclitaxel liposomes using focused ultrasound with microbubbles for treating nude mice bearing intracranial glioblastoma xenografts. Int. J. Nanomed..

[179] Arvanitis C.D., Askoxylakis V., Guo Y., Datta M., Kloepper J., Ferraro G.B., Bernabeu M.O., Fukumura D., McDannold N., Jain R.K. (2018). Mechanisms of enhanced drug delivery in brain metastases with focused ultrasound-induced blood-tumor barrier disruption. Proc. Natl. Acad. Sci USA.

[180] Pluen A., Boucher Y., Ramanujan S., McKee T.D., Gohongi T., di Tomaso E., Brown E.B., Izumi Y., Campbell R.B., Berk D.A. (2001). Role of tumor-host interactions in interstitial diffusion of macromolecules: Cranial vs. subcutaneous tumors. Proc. Natl. Acad. Sci USA.

[181] Netti P.A., Hamberg L.M., Babich J.W., Kierstead D., Graham W., Hunter G.J., Wolf G.L., Fischman A., Boucher Y., Jain R.K. (1999). Enhancement of fluid filtration across tumor vessels: Implication for delivery of macromolecules. Proc. Natl. Acad. Sci USA.

[182] Mainprize T., Lipsman N., Huang Y., Meng Y., Bethune A., Ironside S., Heyn C., Alkins R., Trudeau M., Sahgal A. (2019). Blood-Brain Barrier Opening in Primary Brain Tumors with Non-invasive MR-Guided Focused Ultrasound: A Clinical Safety and Feasibility Study. Sci. Rep..

